# Acupoint Herbal Patching for Asthma: A Systematic Review and Meta-analysis of Randomized Controlled Trials

**DOI:** 10.1097/MD.0000000000002439

**Published:** 2016-01-15

**Authors:** Sun Haeng Lee, Gyu Tae Chang, Xiuyu Zhang, Hyangsook Lee

**Affiliations:** From the Department of Clinical Korean Medicine, Graduate School, Kyung Hee University, Seoul, Korea (SHL, GTC); Department of Pediatrics, Korean Medicine Hospital, Kyung Hee University Medical Center, Seoul, Korea (SHL); Department of Pediatrics of Korean Medicine, Kyung Hee University Hospital at Gangdong, Seoul, Korea (GTC); Acupuncture and Meridian Science Research Center, College of Korean Medicine, Kyung Hee University, Seoul, Korea (XZ, HL); and Department of Korean Medical Science, Graduate School, Kyung Hee University, Seoul, Korea (XZ, HL).

## Abstract

Acupoint herbal patching (AHP), which involves local point stimulation with a herbal medicine patch, has long been used to treat patients with asthma in East Asian countries. However, its evidence is equivocal. This systematic review aims to summarize and critically evaluate the efficacy and safety of AHP for asthma.

A literature search was conducted in PubMed, EMBASE, the Cochrane library, and the China National Knowledge Infrastructure for studies published on or before April 2014, which were randomized controlled trials (RCTs) examining AHP therapy by itself or in combination with other treatments in asthma patients. Trials needed to report pulmonary function outcomes to be included in analyses. The risk of bias of included studies was assessed using the Cochrane risk of bias assessment tool. For statistical pooling, risk ratio, mean difference (MD), or standardized MD was calculated with 95% confidence intervals (CIs) in a random-effects model.

We ultimately included 16 RCTs with 1287 asthmatic patients in analyses. Treatment with AHP improved forced expiratory volume in 1 second (FEV1) by 13% (MD = 12.99%, 95% CI 5.17%–20.81%) and asthmatic symptoms by 60% (risk ratio of unchanged or getting worse symptoms with AHP = 0.4, 95% CI 0.27–0.58) over that observed with placebo. However, evidence is limited due to the heterogeneity and paucity of data. When added to conventional therapies, AHP significantly improved the FEV1/forced vital capacity ratio by 11.6% (95% CI 8.49%–14.79%) and reduced the risk of asthmatic symptoms by 69% (95% CI 0.16–0.58). Compared with conventional medication, AHP significantly improved FEV1 (standardized MD = 0.46, 95% CI 0.05–0.87), but a substantial heterogeneity was detected (*I*^2^ = 53%). When added to Chinese herbal medicine, there were no additional benefits of AHP on pulmonary function or global symptom improvement. No serious adverse events were associated with AHP.

Evidence for AHP efficacy is encouraging, but not conclusive, because of clinical diversity and the high risk of bias in the examined studies. Further clinical and basic research is needed to determine the role of AHP in lung function and symptom improvement in patients with asthma.

## Abstract 2

Supplemental Digital Content is available in the text

## INTRODUCTION

Asthma is a heterogeneous disease that is usually characterized by chronic airway inflammation. It is defined by a history of respiratory symptoms, including wheezing, shortness of breath, chest tightness, and coughing, which vary over time and in intensity, and are accompanied by variable expiratory airflow limitations.^1^ With approximately 300 million people affected,^2^ the global prevalence of asthma ranges from 1% to 16%.^3^ Additionally, asthma symptom prevalence in children continues to rise in regions with a previously low incidence.^4^ Asthma is clinically diagnosed by symptoms of airflow limitation after allergen exposure and by family history of asthma or atopic disease. Current therapeutic strategies for asthma are based on severity and symptom control. Inhaled corticosteroids (ICS) are the mainstay of long-term treatments for persistent asthma,^1^ but approximately 10% of asthmatic patients have refractory disease, despite optimal therapy, leading to increased morbidity and treatment costs.^5^ Therefore, novel treatment approaches or adjunct therapies are necessary for both inflammation and bronchoconstriction in patients with steroid-dependent or steroid-resistant asthma.^6,7^

Acupoint herbal patching (AHP) involves externally applying a processed herbal medicine preparation patch to acupoints or specific sites on the body.^8^ Acupoints have been defined as specific sites through which body organs and meridians qi is transported.^9^ In East Asian countries, AHP has been widely used for a variety of conditions, including asthma, particularly in children.^10,11^ Previous studies have demonstrated that AHP reduces airway inflammation and effectively prevents and treats asthma symptoms, possibly by regulation of serum immunoglobulin E (IgE), eosinophils, nitric oxide, T-lymphocyte subsets, and acetylcholine.^12,13^ However, evidence from AHP study reviews remains inconclusive on whether or not asthma symptoms improve with the treatment, likely because of different research methodologies, heterogeneous interventions, and subjective outcome measures.^14–16^ Additionally, Wechsler et al^17^ examined the effect of a placebo treatment and showed a clinically meaningful improvement in patient-reported subjective symptoms. However, objective outcomes, including forced expiratory volume in 1 second (FEV1), were not affected in asthmatic patients. Given that the majority of clinical studies examining AHP for asthma used patient-reported symptom improvements as a main outcome, the need to properly evaluate AHP efficacy in patients with asthma has emerged. Therefore, high-quality studies using objective outcome measures, such as pulmonary function, are needed. In this context, we conducted a systematic review and meta-analysis of randomized controlled trials (RCTs) reporting objective outcomes of pulmonary function. We use this review to critically summarize and evaluate current evidence on the efficacy and safety of AHP for treating asthma.

## METHODS

### Literature Search Strategy

Search terms and databases were determined through discussion among all authors before searching literature databases. One author (S.H.L.) performed electronic literature searches in PubMed, EMBASE, the Cochrane library, and the China National Knowledge Infrastructure (CNKI) databases to identify RCTs of AHP for asthma. All studies were published on or before April 2014. All databases were comprehensively searched without using filters or language restrictions. For PubMed, EMBASE, and the Cochrane library, search terms were modified from search methods of the Cochrane Airways Group (Supplement 1). The following search terms were used for CNKI: (“chuan” OR “chuanxi” OR “xiaochuan”) AND (“Tie” OR “Tiefu” OR “Futie” OR “Xueweitiefu” OR “Tianjiu” OR “Fapaojiu” OR “Fujiu” OR “Fujiujiu” OR “Sanfujiu” OR “Dongbingxiazhi” OR “Dongbingdongzhi”). The former terms are Chinese for asthma and the latter terms are Chinese for AHP. All articles were screened by 1 reviewer (S.H.L.) and checked by another (X.Z.). Disagreements between reviewers were resolved by discussion with the corresponding author of this review (H.L.). We contacted the corresponding authors of included publications by e-mail or phone if additional information was needed.

### Inclusion/Exclusion Criteria

We only included RCTs that clearly described the method of random sequence generation. If such information was not available or clear in the publication, we contacted the first or corresponding author by e-mail or phone for additional information on randomization. Articles with no response to multiple e-mails or phone calls were excluded since recent research indicated that a large proportion of Chinese RCTs were not truly randomized.^18^ Studies involving adults and/or children were considered for inclusion in analyses if participants were diagnosed with asthma based on clinical symptoms and physiologic features (eg, Global Initiative for Asthma [GINA] criteria).^19^ Studies of asthma on patients with an underlying pulmonary disease (eg, chronic obstructive pulmonary disease, bronchiolitis, bronchitis, and pneumonia) or that used controversial diagnostic categories (eg, asthmatic bronchitis and cough variant asthma) were excluded from analyses.^20^ Treatment with AHP was defined as the use of a herbal preparation patch that covered acupoints for a certain period of time. Studies comparing AHP as an adjunct or sole intervention to active treatments or a placebo were included. Study outcome measures had to include at least one of the following pulmonary function tests: FEV1, forced vital capacity (FVC), FEV1/FVC, or peak expiratory flow (PEF).

### Data Extraction and Risk of Bias Assessment

Data were extracted using a predefined form and checked by 2 independent reviewers. Items on the form included author, publication year, sample size, patient age, participant diagnosis, experimental and control intervention details, and outcomes. Some extracted pulmonary function data were standardized to enable comparisons across studies. Because intervention and control procedures were expected to greatly vary across trials, studies were categorized and summarized according to the type of control interventions. Attempts were made to obtain further details from the original studies’ first or corresponding authors when data were incomplete or missing.

A risk of bias for the included studies was evaluated by the 2 independent reviewers (S.H.L. and H.L.) using the Cochrane risk of bias assessment tool.^21^ Each study was assessed for random sequence generation and allocation concealment (selection bias), blinding of participants and personnel (performance bias), blinding of outcome assessment (detection bias), incomplete outcome data reporting (attrition bias), and selective outcome reporting (reporting bias). Each risk of bias item was given Y (yes), N (no), or U (unclear), where Y indicates low risk of bias, N indicates high risk of bias, and U indicates unclear risk of bias. Disagreements were resolved by discussion with other reviewers.

### Statistical Analyses

The Review Manager software from the Cochrane Collaboration (version 5.2.11 for Windows, The Nordic Cochrane Centre, Copenhagen, Denmark) was used to perform for statistical analyses. In our meta-analysis, only studies reporting similar outcome measures were combined by the type of control interventions. Studies with significant clinical heterogeneity were excluded when data were pooled. A random-effects model was used to estimate the treatment effect because a high variability in AHP effect was expected. This model gives more weight to smaller studies than a fixed-effect model if heterogeneity occurs.^22^ The impact of AHP on dichotomous outcomes was expressed as risk ratio (RR) with 95% confidence intervals (CIs). For continuous outcomes, AHP impact was examined using standardized mean difference (SMD) or mean difference (MD) with 95% CI. Visual inspection of forest plots and *I*^2^ statistics were used to evaluate heterogeneity. An *I*^2^ value of 50% or more was considered to be an indicator of substantial heterogeneity.^23^ To evaluate whether the findings were affected, sensitivity analyses were carried out among studies that had a low risk of a selection bias. In a previous study, children younger than 19 years of age were more likely to report that AHP was effective.^24^ Therefore, we also explored whether the effect of AHP was different between children and adults using a subgroup analysis.

### Ethical Review

This systematic review of already published data did not require ethics committee approval or patient consent.

## RESULTS

### Study Selection

**FIGURE 1:**
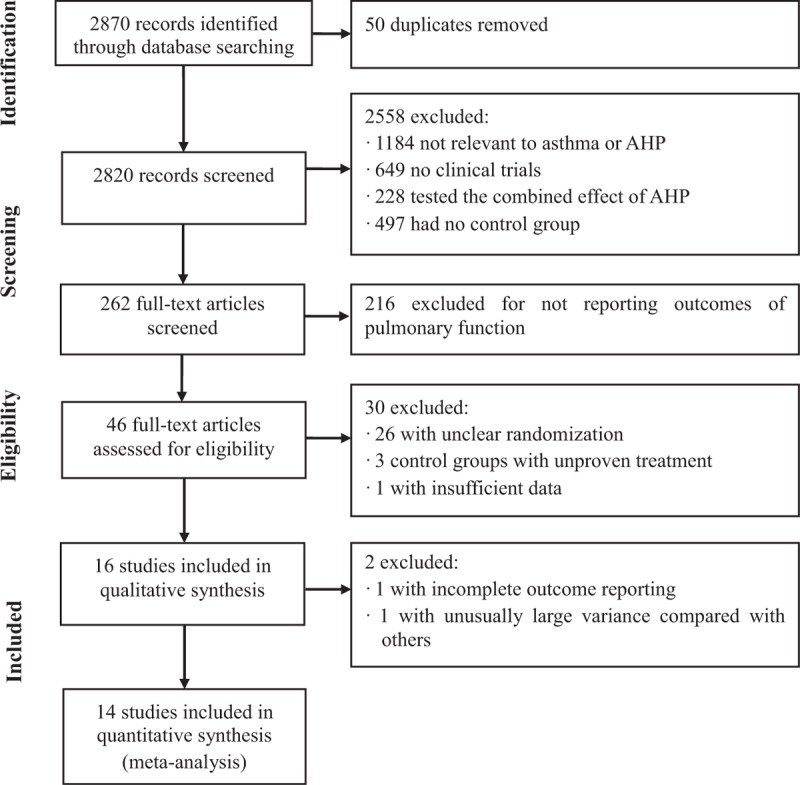
Preferred Reporting Items for Systematic Reviews and Meta-Analyses (PRISMA) diagram of searching. AHP = acupoint herbal patching.

A total of 2870 studies were initially retrieved, with 2613 studies in CNKI, 145 studies in EMBASE, 84 studies in the Cochrane library, and 28 studies in PubMed. After removing 50 overlapping articles, 2820 studies were screened for eligibility. Among these, 2558 studies were excluded based on the title and abstract. After reading the full text of each article, 216 studies were excluded because no pulmonary function data were reported. Of the remaining 46 eligible studies, 15 studies^25–39^ reported adequate randomization and were included in analyses. Of the remaining 31 studies, 1 author (X.Z.) contacted the first or corresponding authors for clarification on randomization and allocation procedures. A total of 10 authors were e-mailed and 9 did not reply. These 9 studies were excluded from analyses. One adequately randomized study was excluded because it did not report the standard deviations of pulmonary function data.^40^ Four corresponding authors were contacted by phone. One study^41^ was included because random numbers were used to determine treatment assignment. Another study^42^ was excluded because the corresponding author did not recall the randomization process. Two corresponding authors^43,44^ refused to reveal the randomization methods. The remaining 17 studies for which the corresponding authors could not be reached were also excluded. Ultimately, 16 Chinese RCTs involving 1287 participants were included in analyses (Figure 1).^45^ Meta-analyses were performed using data from 14 studies involving 1186 participants. One study^29^ only reported PEF data for 50 of 87 children and the other study^37^ had unusually large variances compared with those from other similar studies. This made it impossible to include these studies in pooled data analyses.

### Study Characteristics

Characteristics of included trials are summarized in Table 1  . All participants were diagnosed using modified GINA criteria.^46,47^ Three studies enrolled more than 100 participants,^25,32,35^ with the remaining studies including between 37 and 100 participants. Four trials^26,29,36,39^ recruited only children, 10 studies^25,27,30–35,37,41^ recruited only adults, and 2 studies^28,38^ recruited both children and adults.

**TABLE 1:**
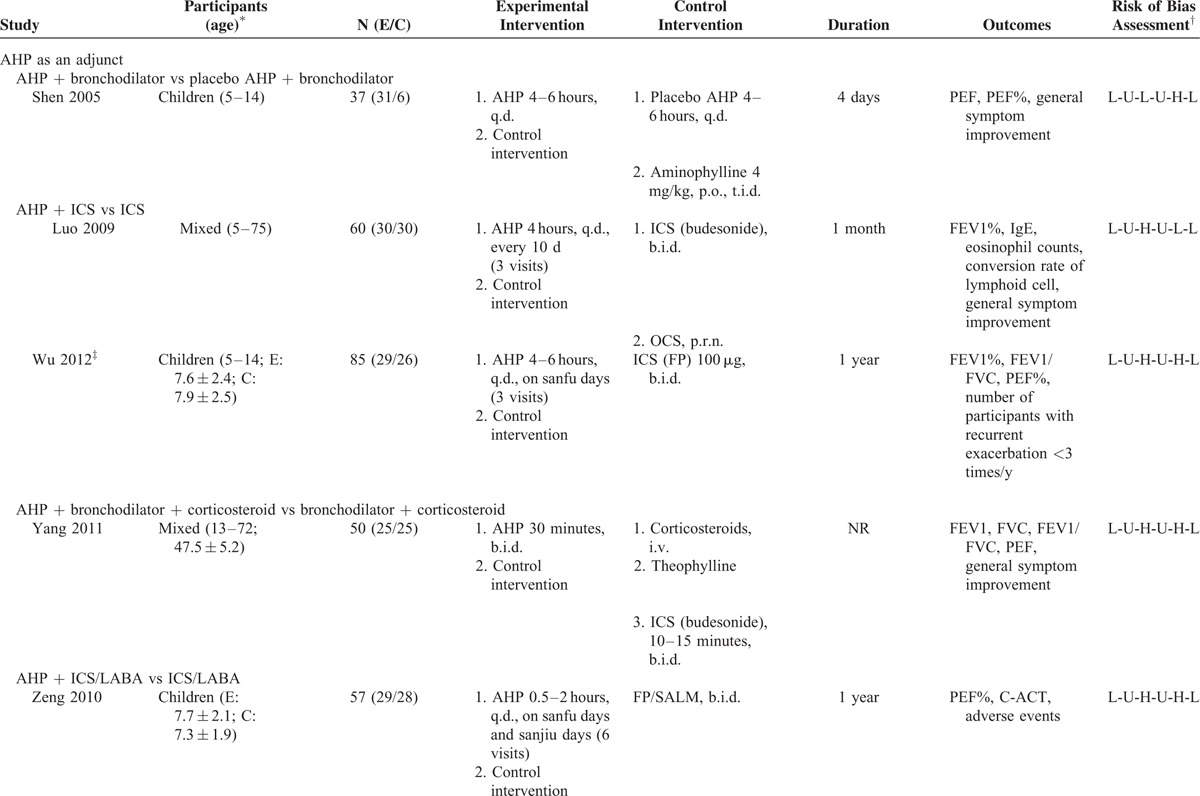
Characteristics of the Included Trials of Acupoint Herbal Patching for Asthma

**TABLE 1 (Continued):**
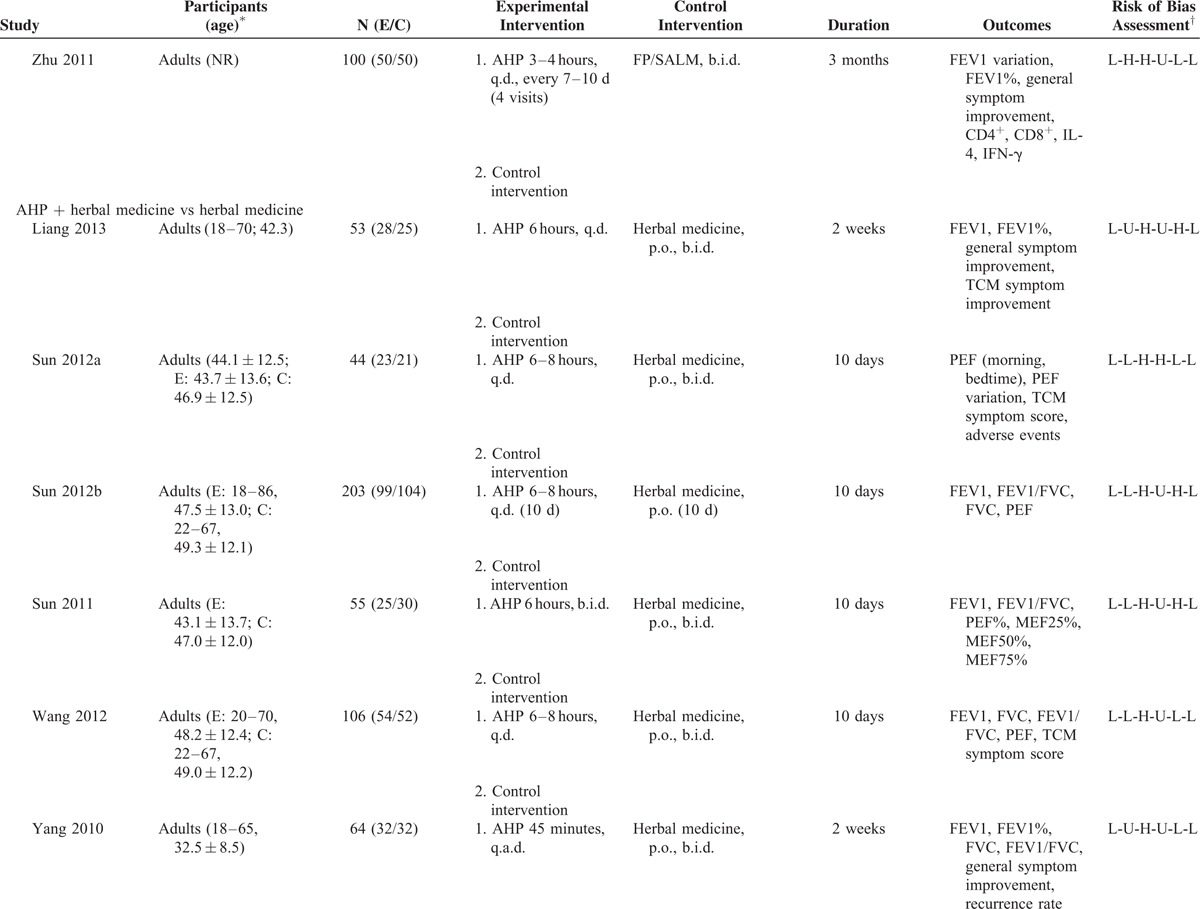
Characteristics of the Included Trials of Acupoint Herbal Patching for Asthma

**TABLE 1 (Continued):**
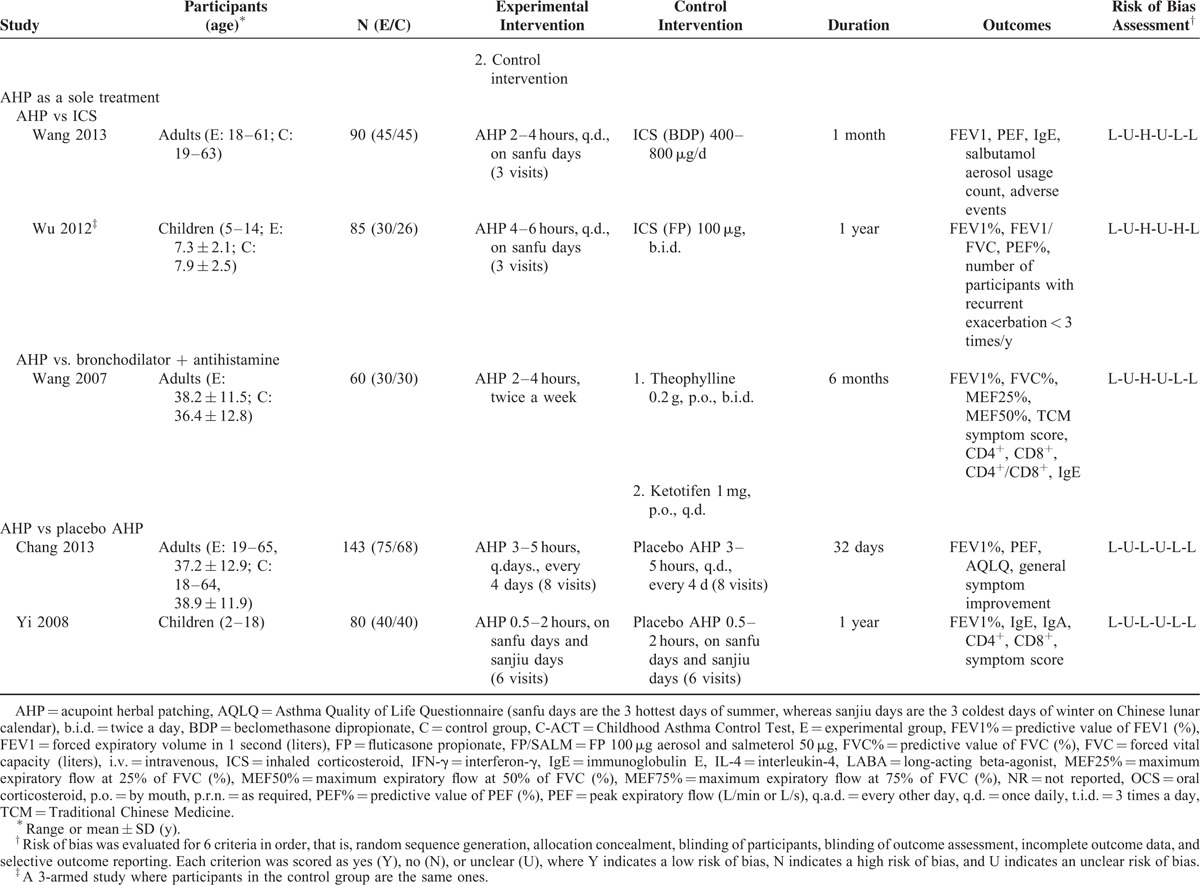
Characteristics of the Included Trials of Acupoint Herbal Patching for Asthma

Treatment with AHP was administered between 3 and 14 times over a period of 4 days to 1 year. Approximately one-third of studies had treated patients with AHP during the San Fu period (between mid-July and mid-August), the hottest time of the year. The most commonly used herbs included *Sinapis alba* (a.k.a. white mustard seeds) and ginger juice, which were used in patches in approximately half of the studies. Half of the included studies did not adequately describe AHP herbs, which prevented proper evaluation of intervention validity.^29–32,34–36,38^ The acupoint BL13, located in the upper back, is associated with lung function and was used in all studies (Supplement 2). Five studies evaluated AHP as an adjunct to conventional medications,^28,29,38,39,41^ 6 studies tested AHP with Chinese herbal medicine against Chinese herbal medicine alone,^27,30–32,35,37^ 2 studies compared AHP alone with active treatment,^33,34^ and 2 studies compared AHP to placebo AHP.^25,26^ One study had 3 treatment arms that compared active treatment with AHP, active treatment alone, and AHP alone.^36^ Pulmonary function measurements were reported in 2 studies as FEV1 in liters and in % predicted.^27,37^ The PEF was measured by either the expert or the participant before intervention in all studies. Morning PEF was preferred for analyses.^30^

### Risk of Bias

**FIGURE 2:**
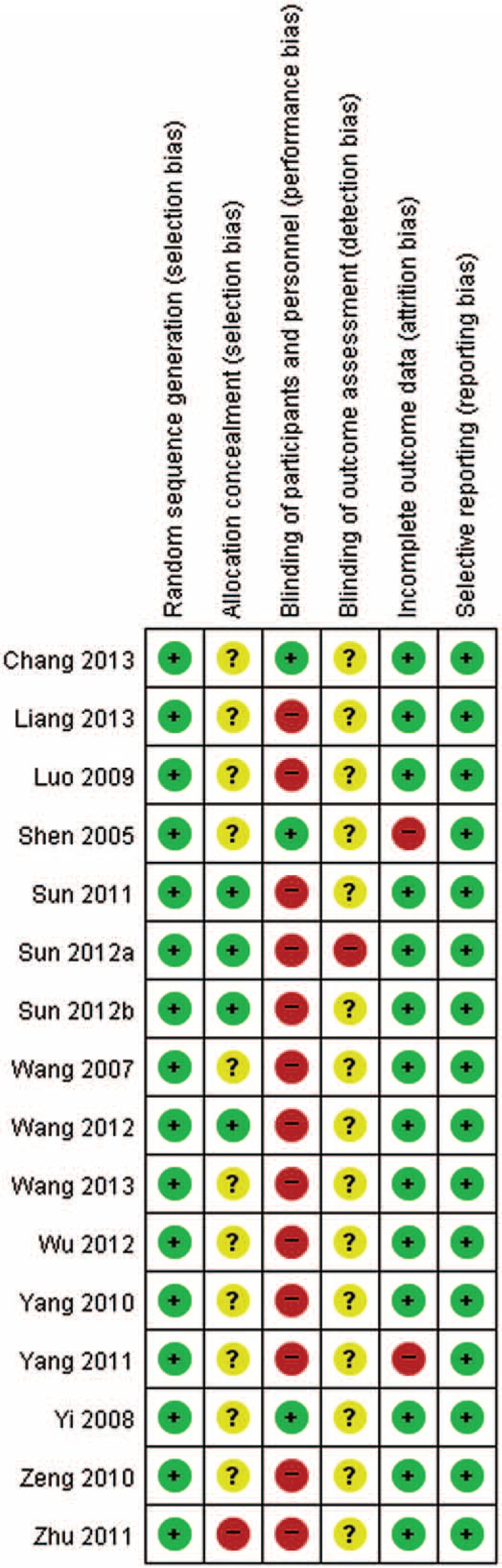
Risk of bias assessed using the Cochrane “Risk of bias” tool. + = low risk of bias, ? = unclear risk of bias, − = high risk of bias.

All but 2^25,26^ of the included trials had an unclear or high risk of bias for more than 1 item (Figure 2). All studies specified the method of randomization. Four trials that centrally randomized participants were given a low risk of bias for allocation concealment,^30–32,35^ but 1 trial was given a high risk of bias based on communication with the corresponding author (ie, study authors were not blinded to group allocation).^41^ Only 3 studies using placebo AHP were given a low risk of bias for participant and outcome assessment blinding.^25,26,29^ Studies that had incomplete outcome data were given a high risk of bias when ≥20% of the participants were missing pulmonary function measurements.^29,38^ No study was determined to have a high risk of bias for selective outcome reporting and significant baseline differences between groups.

### Efficacy of Acupoint Herbal Patching

Data from 14 studies involving 1186 participants were included in this analysis. We summarized the outcomes of the included trials based on the following 3 treatment categories: AHP versus placebo, AHP versus medication, and (3) AHP as an adjunct to other treatments.

#### Acupoint Herbal Patching Versus Placebo

**FIGURE 3:**
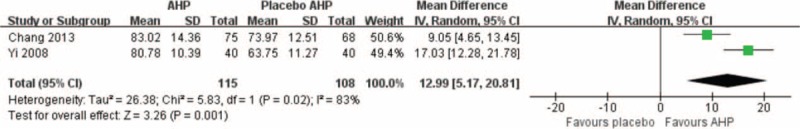
Effect of acupoint herbal patching (AHP) versus placebo on forced expiratory volume in 1 second (FEV1).

Studies comparing AHP to a placebo AHP have been performed in both adults^25^ and children.^26^ Eight AHP treatment sessions were administered over 1 month in adults,^25^ and 6 AHP treatment sessions were administered over 1 year in children.^26^ Mean FEV1 in the AHP group was approximately 13% higher than in the placebo group (2 studies, n = 223 patients, MD = 12.99%, 95% CI 5.17–20.81%).^25,26^ However, there was a substantial heterogeneity between studies (*χ*^2^ = 5.83, *P* = 0.02, *I*^2^ = 83%; Figure 3). In adults, AHP had significant improvements over placebo in PEF (1 study, n = 143 patients, MD = 17.23 mL/s, 95% CI 11.94–22.52 mL/s) and quality of life (1 study, n = 143 patients; MD of Asthma Quality of Life Questionnaire = −42.89, 95% CI −58.13 to −27.65).^25^ Additionally, AHP in adults reduced the risk of unchanged or worsening asthma symptoms by 60% compared with what was observed in the placebo group (1 study, n = 143 patients; RR of asthma symptoms unchanged or worsening with AHP = 0.4, 95% CI 0.27–0.58).^25^

#### Acupoint Herbal Patching Versus Medication

**FIGURE 4:**
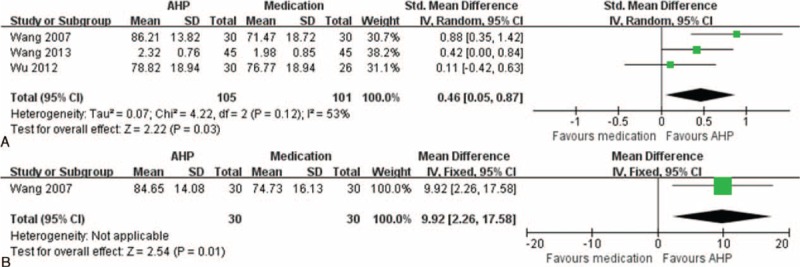
Effects of acupoint herbal patching (AHP) versus medication on forced expiratory volume in 1 second (FEV1, A) and forced vital capacity (FVC, B).

Acupoint herbal patching was compared with ICS in 2 studies^33,36^ and with a bronchodilator and an antihistamine in 1 study.^34^ The FEV1 was significantly higher in the AHP groups than in the medication groups (3 studies, n = 206 patients; SMD = 0.46, 95% CI 0.05–0.87, *I*^2^ = 53%; Figure 4A).^33,34,36^ The FVC also significantly improved 6 months after initiating AHP therapy in comparison with bronchodilator and antihistamine therapy (1 study, n = 60 patients, MD = 9.92%, 95% CI 2.26–17.58%; Figure 4B).^34^ However, a superior effect of AHP was not demonstrated for other outcome measures, including PEF (2 studies, n= 146 patients, SMD = 0.12, 95% CI −0.43 to 0.68, *I*^2^ = 63%)^33,36^ and FEV1/FVC (1 study, n = 56 patients, MD = −2.88%, 95% CI −13.07 to 7.31%).^36^

#### Acupoint Herbal Patching as an Adjunct to Other Interventions

**FIGURE 5:**
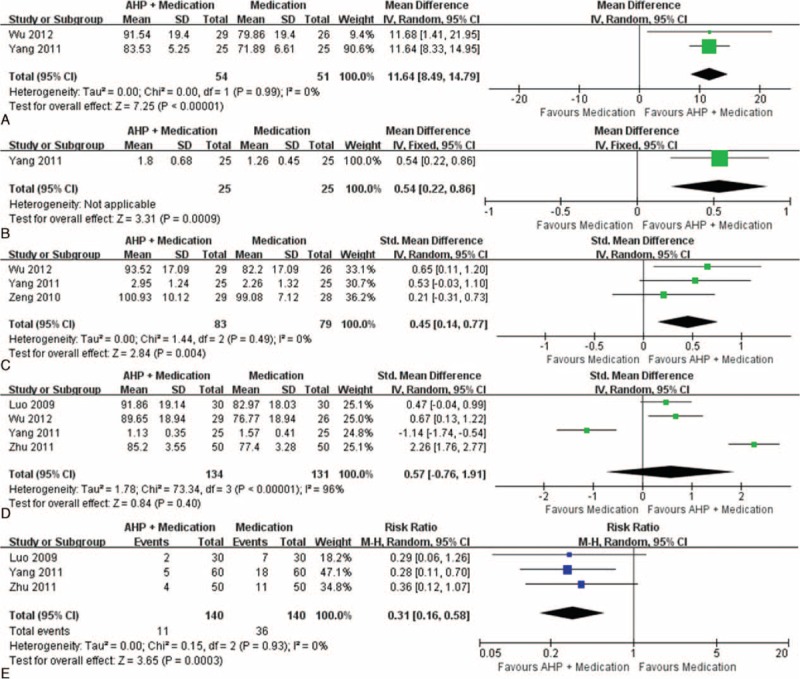
Effects of acupoint herbal patching (AHP) with medication versus medication alone on FEV1/FVC (A), FVC (B), PEF (C), FEV1 (D), and risk of symptom persisting with treatment (E). FEV1 = forced expiratory volume in 1 second, FVC = forced vital capacity, PEF = peak expiratory flow.

Twelve studies examined if AHP improves pulmonary function when added to conventional^28,29,36,38,39,41^ or Chinese herbal^27,30–32,35,37^ medications. When AHP was added to conventional medical therapies, significant improvements were observed in FEV1/FVC (2 studies, n = 105 patients, MD = 11.64%, 95% CI 8.49–14.79%, *I*^2^ = 0%; Figure 5A),^36,38^ FVC (1 study, n = 50 patients, MD = 0.54 L, 95% CI 0.22–0.86 L; Figure 5B),^38^ and PEF (3 studies, n = 162 patients, SMD = 0.45, 95% CI 0.14–0.77, *I*^2^ = 0%; Figure 5C).^36,38,39^ However, no significant differences were observed in either FEV1 (4 studies, n = 265 patients, SMD = 0.57, 95% CI −0.76 to 1.91, *I*^2^ = 96%; Figure 5D)^28,36,38,41^ or the Childhood Asthma Control Test (1 study, n = 57, MD = 0.38, 95% CI −0.13 to 0.89).^39^ The number of patients that had a risk of persistent asthma symptoms in the AHP and medication group was approximately 31% of that in the medication-only group (3 studies, n = 280 patients, RR = 0.31, 95% CI 0.16–0.58, *I*^2^ = 0%; Figure 5E).^28,38,41^ When AHP was administered as an adjunctive therapy to herbal medicine, none of the following outcomes were improved: FEV1 (4 studies, n = 417 patients, SMD = −0.27, 95% CI −0.94 to 0.40, *I*^2^ = 90%),^27,31,32,35^ FEV1/FVC (%) (3 studies, n = 364, MD = −1.69%, 95% CI −4.57 to 1.19%, *I*^2^ = 18%),^31,32,35^ FVC (2 studies, n = 309 patients, MD = −0.10 L, 95% CI −0.31 to 0.11 L, *I*^2^ = 0%),^32,35^ PEF (4 studies, n = 408 patients, SMD = −0.12, 95% CI −0.44 to 0.21, *I*^2^ = 58%),^30–32,35^ and global symptom improvement (1 study, n = 60 patients, RR = 0.29, 95% CI 0.06–1.26).^27^

### Sensitivity Analyses

Sensitivity analyses were conducted to explore whether outcomes were affected by limiting the included studies to those with a low risk of bias for allocation concealment.^30–32,35^ All 4 trials that had a low risk tested AHP as an adjunct therapy to Chinese herbal medicine. Our analyses described above detected no benefit of AHP in any outcomes when AHP therapy was added to herbal medicine. Our sensitivity analysis confirmed no additional benefit of AHP on FEV1 (3 studies, n = 364 patients, SMD = −0.52, 95% CI −1.15 to 0.11, *I*^2^ = 87%),^31,32,35^ FEV1/FVC (%) (3 studies, n = 364 patients, MD = −1.69%, 95% CI −4.57 to 1.19%, *I*^2^ = 18%),^31,32,35^ FVC (2 studies, n = 309 patients, MD = −0.10 L, 95% CI −0.31 to 0.11L, *I*^2^ = 0%),^32,35^ and PEF (4 studies, n = 408 patients, SMD = −0.12, 95% CI −0.44 to 0.21, *I*^2^ = 58%).^30–32,35^

### Subgroup Analysis

A planned subgroup analysis on adults and children was not performed because there were too few studies to perform a meaningful comparison.

### Safety

Reported adverse reactions associated with AHP included skin redness^30,39^ and local itching.^30,33^ However, these symptoms subsided by removing the patch, keeping it dry, and reducing wearing time. The frequency of reported adverse events was low (2 of 23 patients [9.7%]^30^ and 4 of 45 patients [8.9%]).^33^ In 1 study, a control group using beclomethasone dipropionate aerosol reported *Candida albicans* stomatitis and hoarseness, and these reported symptoms were treated with clotrimazole and NaHCO_3_ gargling (3 of 45 patients [6.7%]).^33^

## DISCUSSION

The purpose of this systematic review and meta-analysis was to estimate the efficacy and safety of AHP for improving lung function and global symptoms in patients with asthma. Our primary analysis of 16 RCTs showed that, compared with a placebo control, AHP significantly improved several clinical asthma outcomes, including FEV1, PEF, and asthma symptoms. Additionally, AHP was beneficial over conventional medications (eg, ICS and long-acting beta2-agonists [LABA]) for improving FEV1 and FVC. However, AHP showed no additional benefits for PEF and FEV1/FVC. When added to conventional medication, AHP significantly improved FEV1/FVC, PEF, and asthma symptoms. However, when AHP was added to Chinese herbal medications, little additional benefit in pulmonary function was observed. Given the substantial heterogeneity among studies and the small number of studies that met inclusion criteria, the findings of our meta-analysis require careful interpretation. Adverse events associated with AHP were generally mild and spontaneously resolved. Nevertheless, adverse events were poorly reported in the included studies, so the evidence on the safety of AHP needs to be further investigated.

Asthma is a widespread chronic airway inflammatory disease characterized by an unpredictable course.^1^ Whereas the majority of patients with asthma are effectively treated with current standard therapy (eg, high doses of ICS with LABA),^5^ some patients do not adequately respond to standard therapy and need novel treatment approaches or adjunct therapies.^6,7^ AHP has been traditionally and frequently used to treat asthma in East Asian countries, including China and Taiwan.^48,49^ This therapy uses a processed herbal medicine patch that is strategically placed on specific acupoints for a range of diseases, including asthma.^11^ Previously, AHP was reported to reduce asthmatic inflammation by reducing levels of IgE, eosinophil, nitric oxide, interleukin (IL)-4, and tumor necrosis factor-α, and increasing levels of IL-10, CD8+, and interferon (IFN)-γ.^12,50,51^ Furthermore, AHP relieves bronchoconstriction by the antiacetylcholine effect, ß-receptor up-regulation, and transforming growth factor-beta1 reduction.^12^ Given these results, asthmatic symptoms might be ameliorated by AHP by reducing bronchoconstriction and lung inflammation. However, previous reviews on AHP for asthmatic symptom improvement were equivocal, probably because of poor methodology and reporting.^14–16,52^ Our meta-analysis only included adequately randomized studies that mainly analyzed objective measures of lung function. Thus the findings of our review may be more reliable than those obtained in previous reviews.^14–16,52^

The majority of included trials were assessed to have one or more high risks of bias. First, adequate randomization and allocation concealment are crucial to obtain unbiased results.^53,54^ Considering recent research found that a large proportion of Chinese-language RCT reports are not actually randomized,^18^ we only included RCTs for which we could confirm adequate randomization methods directly from the report or from the study authors. Accordingly, the included studies all had a low risk of bias for adequate randomization. However, only a quarter of them had a low risk of bias for adequate allocation concealment.^30–32,35^ Therefore, the included studies are not entirely free from selection biases.

Wechsler et al^17^ recently reported that placebo effects can be clinically meaningful and can rival the effects of active medication in patients with asthma. In our review, only 3 of 16 studies were placebo-controlled.^25,26,29^ Unblinding could bias study outcomes due to different treatment expectations.^21^ Whereas ß-blockers as an adjunct asthma therapy to ICS resulted in methacholine airway hyperresponsiveness (AHR) improvement in open-label studies, a blinded and controlled study showed no significant effect of AHR.^55^ Thus, unblinded studies in our review might also have overestimated the effect of AHP, even when objective outcomes like pulmonary function were examined. A funnel plot asymmetry analysis could not be carried out to detect potential small study effects due to a small number of studies. It is worth noting that Chinese herbal medicine studies published in Chinese-language journals have been shown to be more likely to have reported larger treatment effects, especially in small studies with lower methodological quality.^56^ In our review, we did not have sufficient data to examine the possible effects of small studies. Taken together, the risk of bias described above likely resulted in an overestimation of the benefit of AHP tested in the included trials.

Our systematic review has several limitations. These include publication bias, small sample size, potentially different disease baseline severity, and AHP intervention diversity. First, the included 16 RCTs were all published in Chinese medical journals. Despite every effort to find all relevant RCTs in various databases and reference lists of related articles, our search strategies and selection criteria might not have been comprehensive enough to locate every relevant RCT or to completely remove publication or location biases. Vickers et al^57^ strongly suggested that China is more likely to publish positive trials. Meanwhile, another study suggested that trials published in English-language journals are more likely to show larger effects than those published in non-English–language journals.^58^ However, this finding has not been shown by other studies.^59–61^ Although publication bias was not formally explored because of a small number of trials, our systematic review may not have been entirely free from publication or location biases because it only included Chinese RCTs. Second, the median sample size of the 14 RCTs included in the meta-analysis was 84 patients and no study reported a formal power analysis. Therefore, we cannot guarantee that statistically significant changes in pulmonary function measures and asthma symptoms were definitely a true effect in the included studies. Instead, some statistically significant results may have occurred because of a low power. Third, heterogeneities among studies were observed, as expected. Participants diagnosed with GINA criteria had different asthma severity and baseline lung function as controls that were given different medications (ie, bronchodilator monotherapy, ICS and LABA, or oral corticosteroids add-on when necessary).^1^ This may partly explain some of the observed heterogeneity for lung function outcomes. Lastly, the generalizability of our findings is limited by the diverse AHP interventions used in the included studies. Tested AHP interventions varied greatly in terms of what herbs and acupoints were used, how long treatment lasted for (4 d to 1 y), and how many AHP sessions were administered (3–14 sessions). Therefore, an optimal AHP intervention could not be determined from our review. All of these intervention diversities may have contributed to the inconsistent effect of interventions under the AHP label.

Considering that all included studies were conducted in China, further research is needed outside of China to improve the generalizability and applicability of study results. To do this, several independent researchers should perform studies based on standardized treatment protocols and optimal AHP interventions, which remains to be determined. Additionally, future trials involving a predefined outcome measure and relevant safety data would enable better comparability of future studies and allow the totality of evidence to be better complied. It also remains unclear how AHP works for asthma. Further basic and clinical research is needed to determine if AHP influences inflammatory markers in patients with asthma. Research regarding which asthmatic severity and phenotypes are best treated with AHP is also needed to determine AHP indications. In our initial search, 30 RCTs were excluded because we could not confirm that they were truly randomized studies from the report itself or from the corresponding author. Transparent reporting in accordance with the Consolidated Standards of Reporting Trials Statement (CONSORT)^62^ and Standards for Reporting Interventions in Controlled Trials of Acupuncture (STRICTA)^63^ is urgently needed because AHP is a combined therapeutic modality of acupoints and Chinese herbs.

## CONCLUSIONS

Acupoint herbal patching alone or in combination with standard medication has been shown to significantly improve several measures of pulmonary function and symptoms of asthma, with few adverse effects. However, the current evidence on AHP is insufficient to recommend it to patients with asthma because previous studies had a large amount of clinical diversity and a high risk of biases. Further clinical and basic research is needed to firmly determine the role of AHP in lung function and symptom improvement in patients with asthma.

## Supplementary Material

Supplemental Digital Content
