# Supplementary material for: Acupoint Herbal Patching for Asthma: A Systematic Review and Meta-analysis of Randomized Controlled Trials
Source: Medicine (Baltimore). 2016 Jan 15;95(2):e2439. doi: 10.1097/MD.0000000000002439 (PMC4718253; doi:10.1097/MD.0000000000002439)
Supplement: Supplemental Digital Content [file medi-95-e2439-s001.pdf]

Supplement1 Search strategy for PubMed
#1 (asthma*)
#2 (antiasthma* or anti-asthma*)
#3 (wheez*)
#4 (bronchospas*)
#5 (bronch* near/3 spasm*)
#6 (bronchoconstrict*)
#7 (bronch* near/3 constrict*)
#8 (bronchial* or respiratory or airway* or lung*) near/3 (hypersensitiv* or hyperreactiv* or allerg* or insufficien*)
#9 (dust or mite*) near/3 (allerg* or hypersensitiv*)
#10 (#1 OR #2 OR #3 OR #4 OR #5 OR #6 OR #7 OR #8 OR #9)
#11 (acu*)
#12 (adhesi*)
#13 (applicat*)
#14 (paste*)
#15 (patch*)
#16 (plaster*)
#17 (stick*)
#18 (#12 OR #13 OR #14 OR #15 OR #16 OR #17)
#19 (#11 AND #18)
#20 (#10 AND #19)

Supplement 2 Details of acupoint herbal patching in the included trials

Author /year	Acupoints		Herbs		Treated in San Fu period*
	Trunk	Limbs	Solid	Liquid	
Chang 2013	1st: BL12, BL13, GV14, CV22, EX-B1<br>2nd: BL13, BL20, BL23, BL43, CV17		1st: *Sinapis alba*, *Asarum sieboldi*, *Corydalis turtschaninovii*, *Euphorbia kansui*, *Saposhnikovia divaricata*, *Schizandra chinensis*, *Dryobalanops aromatica* (borneol)<br>2nd: *Sinapis alba*, *Asarum sieboldi*, *Corydalis turtschaninovii*, *Euphorbia kansui*, *Cinnamomum cassia* (cinnamon), *Zingiber officinale* (dried ginger), *Epimedium brevicornum*, *Dryobalanops aromatica* (borneol)	Juice of *Zingiber officinale* (ginger)	Y
Liang 2013	BL13, CV17, CV22		Not reported	Not reported	N
Luo 2009	1st: BL12, BL13, EX-B1<br>2nd: BL14, BL20, GV14<br>3rd: BL11, BL23, BL43		*Euphorbia kansui*, *Sinapis alba*, *Ephedra sinica* (mahuang), *Asarum sieboldi*, *Corydalis turtschaninovii*, *Moschus moschiferus* (musk)	Juice of *Zingiber officinale* (ginger)	N
Shen 2005	BL13, BL43, EX-B1		*Ephedra sinica* (mahuang), *Asarum sieboldi*, *Sinapis alba*, *Ardisia bicolor*, etc.		N
Sun 2012a	BL13, CV17, CV22, EX-B1		*Datura metel*, *Sinapis alba*, *Prunus armeniaca*, *Zingiber officinale* (ginger), etc.		N
Sun 2012b	BL13, CV17, CV22, EX-B1		*Datura metel*, *Sinapis alba*, *Prunus armeniaca*, *Zingiber officinale* (ginger), etc.		N
Sun 2011	BL13, CV17, CV22, EX-B1		*Datura metel*, *Sinapis alba*, *Prunus armeniaca*, *Zingiber officinale* (ginger), etc.**		N
Wang 2013	BL12, BL13, BL17, BL20, BL23, BL43, GV14, EX-B1		*Ephedra sinica* (mahuang), *Asarum sieboldi*, *Euphorbia kansui*, *Corydalis turtschaninovii*, *Sinapis alba*, *Moschus moschiferus* (musk)	Juice of *Zingiber officinale* (ginger)	Y
Wang 2012	BL13, CV17, CV22, EX-B1		*Datura metel*, *Sinapis alba*, *Prunus armeniaca*, *Zingiber officinale* (ginger), etc.		N
Wang 2007	BL11, BL13, BL43, CV22		*Sinapis alba*, *Euphorbia kansui*, *Asarum sieboldi*, *Angelica dahurica*, *Scutellaria baicalensis*, *Eugenia caryophyllata* (clove), *Cinnamomum cassia* (cinnamon), etc.	Juice of *Zingiber officinale* (ginger)	N
Wu 2012	BL13, BL15, BL17		*Sinapis alba*, *Asarum sieboldi*, *Corydalis turtschaninovii*, *Euphorbia kansui*, etc.	Juice of *Zingiber officinale* (ginger)	Y
Yang 2011	BL13, BL20, EX-B1		*Sinapis alba*, *Evodia rutaecarpa*, Foeniculi Fructus, *Asarum sieboldi*, *Dryobalanops aromatica* (borneol), etc		N

Yang 2010	LU7, BL13, BL43, EX-B1		*Sinapis alba*, *Euphorbia kansui*, *Corydalis turtschaninovii*, *Asarum sieboldi*, *Pinellia ternata*	Juice of *Zingiber officinale* (ginger)	N
Yi 2008	BL13, BL43, CV17, EX-B1		*Sinapis alba*, *Asarum sieboldi*, *Moschus moschiferus* (musk), *Euphorbia kansui*, *Corydalis turtschaninovii*	Juice of *Zingiber officinale* (ginger)	Y
Zeng 2010	BL13, BL20, BL23, EX-B1		*Capsicum annuum* (chili pepper), *Cinnamomum cassia* (cinnamon), *Zingiber officinale* (ginger)		Y
Zhu 2011	BL13, BL23, GV12, CV22, EX-B1	ST36, ST40[†]	*Mylabris phalerata, Sinapis alba*		N

[*] In Chinese Lunar Calendar, San Fu refers to the hottest period of the year between mid-July to mid-August. This period is considered important as Yang qi is the strongest according to Yin-Yang theory in traditional Chinese Medicine.

[**] Specific herbs were not reported but prescription was same with other Sun's report.

[†] ST40 was selected in case of need.
